# Age and manifestation related symptoms in familial adenomatous polyposis

**DOI:** 10.1186/1471-2407-5-24

**Published:** 2005-03-02

**Authors:** Roland S Croner, Wolfgang M Brueckl, Bertram Reingruber, Werner Hohenberger, Klaus Guenther

**Affiliations:** 1 Department of Surgery, University of Erlangen, Maximilliansplatz 1, D-91054 Erlangen, Germany; 2 Department of Internal Medicine I, University of Erlangen, Ulmenweg 18, D-91054 Erlangen, Germany

## Abstract

**Background:**

To identify early symptoms of familial adenomatous polyposis with a view to improve early diagnosis and treatment. Diagnosis on the basis of genetic testing is usually limited to where there is a known family history, so FAP is more usually diagnosed on clinical grounds. Except for those identified via FAP registers, the majority of patients are symptomatic at the time of diagnosis.

**Methods:**

We undertook a retrospective study of 143 FAP patients treated at the Department of Surgery, University of Erlangen between 1971 and 2000. We identified patterns of symptoms, endoscopic findings and extracolonic manifestations in three age groups.

**Results:**

FAP was diagnosed clinically on the basis of symptoms in 84% (120/143) of these patients. Most presented with intestinal symptoms such as colonic bleeding (68%) and diarrhea (42%). All but one of the patients between 20 and 40 years old had rectal polyps (98.7%, 75/76), whereas in those over 40 years old the prevalence was 76% (35/46). Non-specific symptoms such as abdominal pain, fatigue and bloating were less frequent and were mainly reported by patients older than 40.

**Conclusion:**

The commonest presenting features of FAP are alteration of bowel habit and rectal bleeding, but both are found in many other conditions. Patients with these findings need immediate endoscopy to allow prompt diagnosis and prophylactic surgery.

## Background

Familial adenomatous polyposis (FAP) is an autosomal dominantly inherited disease and is caused by germline mutations in the adenomatous polyposis coli gene (APC) in chromosome 5q21 [1]. Somatic mutations of the APC gene occur in about 80% of sporadic colorectal cancers. APC encodes for a multimodal protein that plays an important role in the wnt-signalling pathway and in intercellular adhesion [2,3]. The APC germline mutation has a penetrance which is close to 100% [4]. Untreated, the disease usually leads to the appearance of hundreds of adenomatous polyps in the colorectum between puberty and age 20 and to cancer by the early forties at the latest which is the most frequent reason for death in patients with FAP [5]. Attenuated forms of FAP (AFAP) are variations in phenotype. AFAP with less than 100 adenomatous polyps is diagnosed at a mean age of 44 years, and cancer is diagnosed at a mean age of 56 years [6]. Congenital hypertrophy of retinal pigment epithelium, upper gastrointestinal polyps, desmoid tumors, adrenal adenomas and osteomas are extracolonic FAP phenotypic features whose expression depends upon the mutated region of the APC gene [7-11].

The incidence of FAP is approximately 1 in 8000 [12]. Early detection, prophylactic surgery and lifelong surveillance are essential to prevent the development of colorectal cancer [13]. When an index patient is newly diagnosed in a center with an established FAP register, screening of family members with molecular diagnostics is carried out as a matter of routine and can detect the condition before the development of symptoms. It is rare for patients outside this system to be diagnosed prior to the development of symptoms. Also in patients with spontaneous new mutations of the APC gene, early clinical diagnosis can increase their descendants' chance of survival. Lack of awareness and the absence of systematic family screening may cause diagnosis to be delayed in patients at risk of inherited colorectal cancer [14]. According to the literature, new mutations occur in 25% of all FAP cases and this predominantly young patient population is burdened with a high risk of colorectal cancer. It is therefore desirable that there are defined clinical algorithms for detecting the characteristic clinical signs of FAP, ideally within a multidisciplinary setting in which patients are entered on an FAP register.

In a retrospective analysis, we evaluated all clinical symptoms of FAP prior to surgery. We categorized these according to patient age and into colorectal and extracolonic manifestations of the disease. Our aim was to detect specific characteristics in each age group which could lead to an early clinical diagnosis and prevent the development of colorectal cancer.

## Methods

### Definition

The diagnosis of FAP was established clinically if more than 100 polyps were detected endoscopically in the colorectum.

### Patient population

Between 1971 and 2000, we registered 157 patients (66 women, 85 men, 6 unknown) with an endoscopic diagnosis of FAP. The mean age at primary diagnosis was 34 (± 15 SD) years with a range of 5 to 73 years. In 151 cases, surgery was performed. Sixty-seven patients had colectomy with ileorectal anastomosis, 55 patients underwent restorative proctocolectomy with ileal pouch-anal anastomosis, and 10 had proctocolectomy with an end-ileostomy. Nineteen patients underwent various other surgical procedures. Presently, 83 patients remain alive and 28 patients have been lost to follow-up. Forty patients are known to have died: 20 of colorectal cancer, 5 due to desmoid tumors, 10 of other causes and 5 for unknown reasons.

A predominant early symptom could be defined and documented in 143 patients. Family screening revealed that 29 of these patients had affected relatives, while no FAP was detected in the families of the remaining 114 patients.

### Evaluation of symptoms

**Figure 1:**
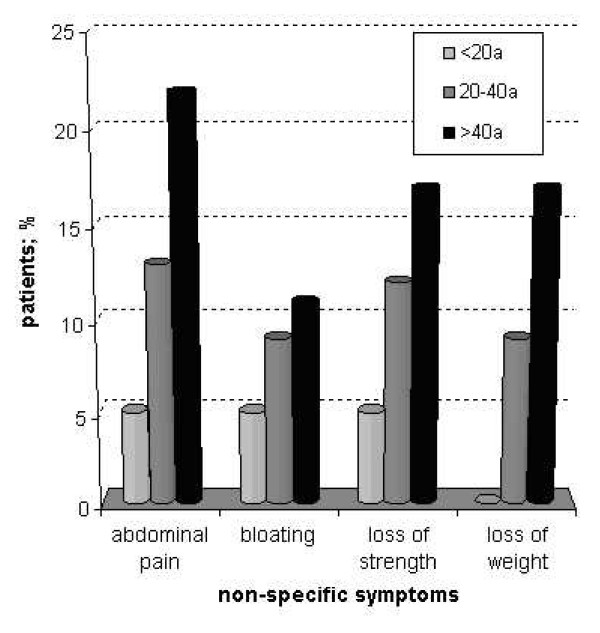
„Non-specific symptoms“ on primary diagnosis of FAP (n = 143 patients, realtive values regarding the corresponding subgroup of age)

**Figure 2:**
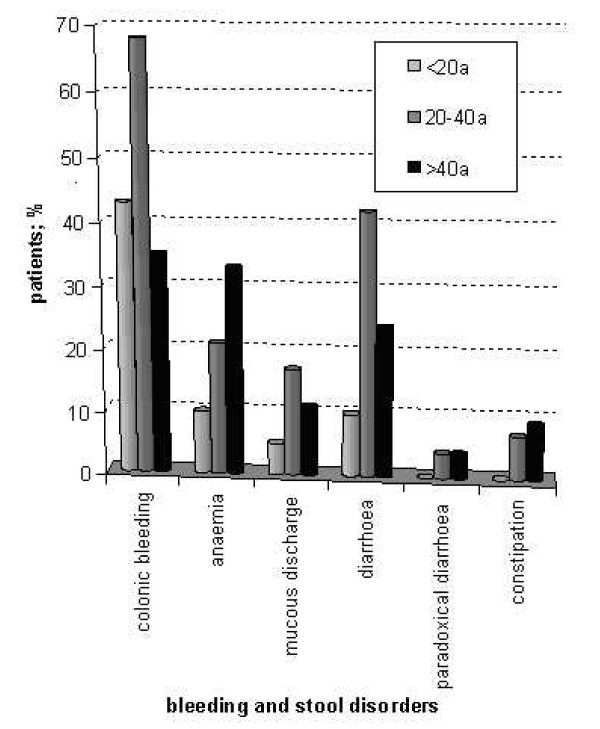
„Bleeding and stool disorders“ on primary diagnosis of FAP (n = 143 patients, realtive values regarding the corresponding subgroup of age)

The predominant symptoms of FAP were analyzed and documented retrospectively in all 143 cases. The entire patient population was divided into three age groups according the time of primary diagnosis: less than 20, 20 to 40, and more than 40 years of age. Abdominal pain, pain during defecation, bloating, fatigue and loss of weight were categorized as non-specific symptoms (figure 1). Colonic bleeding, anemia, mucous discharge, diarrhea, paradoxical diarrhea and constipation were categorized as bleeding and stool disorders (figure 2).

### Evaluation of colorectal and extracolonic manifestations

The endoscopic reports were obtained and the pattern of polyps in the colorectum was divided into several groups: no rectal polyps, less than 10 rectal polyps, 10–100 polyps, and more than 100 polyps. We recorded the prevalence of carcinoma at the primary FAP diagnosis (table 1). In addition, we documented any extracolonic manifestations of FAP: desmoid tumors (abdominal wall, mesenteric, retroperitoneal), gastric polyps, duodenal polyps and polyps of the small bowel (table 2).

### Data presentation

The relative prevalence of symptoms in each age group is shown in figures 1 and 2. Molecular findings are grouped according to the mutations detected in the APC gene (table 3). Colorectal and extracolonic manifestations at primary diagnosis are presented for each of the recorded symptoms. Values in brackets indicate the percentage of affected patients in each subgroup (tables 1 and 2).

## Results

### Age and symptoms

The diagnosis of FAP was made on the basis of clinical symptoms in 66% of patients (94/143). Diagnosis was incidental in 6% (9/143) and the reasons are unknown in 8% (11/143).

Most patients (53%, 76/143) were between 20 and 40 years of age at primary diagnosis, of whom 75% (58/76) had symptoms. 32% (46/143) of our patients were older than 40 years at primary diagnosis, of whom 65% (30/46) had symptoms. Only 15% of patients (21/143) were less than 20 years old at diagnosis. Within this group five patients were under 10 years old. Only 33% (7/21) of patients under 20 years presented with symptoms.

### Molecular diagnostics

Table 3 shows the results of molecular diagnostics in 42 patients (42/143, 29%). These were carried out in 43% (9/21) of patients less than 20 years old, 28% (21/76) of patients 20–40 years old and 26% (12/46) of patients over 40 years of age. Eighteen out of 42 mutations in the APC gene (42.9%) were detected in exon 15, six (14.3%) were detected in exon 14, and six (14.3%) in exon 5. Exon 3 and exon 10 showed one mutation each (1/42, 2.3%).

### Colorectal manifestations at primary diagnosis of FAP

Table 1 shows the colorectal findings at primary diagnosis, when 63% (90/143) of our patients had more than 100 polyps in the colon and 67% (96/143) between 10 and 100 polyps in the rectum. Among the patients younger than 20 years, 57% (12/21) had more than 100 colonic polyps and 67% (14/21) had between 10 and 100 rectal polyps. Patients between the age of 20 and 40 years had the highest prevalence of rectal polyps. In 79% (60/76) of this age group 10–100 rectal polyps could be detected and only 1.3% (1/76) had no rectal polyps. Among patients over 40 years of age, 24% (11/46) had no rectal polyps but 63% (29/46) had more than 100 colonic polyps.

Carcinomas of the colon were detected in 17% (25/143) and rectal carcinomas in 12% (17/143). No patient younger than 20 years had developed colorectal cancer. In the group between 20 and 40 years old, rectal carcinoma was detected in 21% (10/76) and colon carcinoma in 17% (13/76). In patients older than 40 years of age, 15% (7/143) had rectal carcinoma and 50% (23/46) had colonic carcinoma.

### Extracolonic manifestations at primary diagnosis of FAP

Table 2 shows the extracolonic manifestations at primary diagnosis. Information about the absence or presence of desmoid tumors of the abdominal wall and the mesenterium was aviable in 119 patients and about retroperitoneal desmoids in 143 patients. Desmoid tumors of the abdominal wall were detected in 7% (8/119), mesenteric desmoids in 11% (13/119) and retroperitoneal desmoids in 4% (5/143). Information about the absence or presence of gastric gland polyps was aviable in 84 patients, about duodenal polyps in 79 and about polyps of the small bowel in 32 patients. Gastric gland polyps were found in 46% (39/84), duodenal polyps in 33% (26/79) and polyps of the small bowel in 22% (7/32).

No patients younger than 20 years suffered from desmoid tumors, but in 20% (3/15) gastric gland polyps and in 13% (2/15) duodenal polyps were discovered. The highest incidence of desmoid tumors (29%, 18/62) was found in the group between 20 and 40 years of age (8% abdominal wall, 15% mesenteric, 5% retroperitoneal). In two cases within this age group the diagnosis of FAP was incidental because the patient presented with a desmoid tumor of the abdominal wall. In patients between 20 and 40 years, gastric gland polyps were detected in 52% (27/52), duodenal polyps in 31% (16/52) and polyps of the small bowel in 14% (4/29). Only 14% (5/36) of patients older than 40 years had desmoid tumors at primary diagnosis of FAP (8% abdominal wall, 8% mesenteric, 2% retroperitoneal). And in this age group 53% (9/17) had gastric gland polyps, 67% (8/12) had duodenal polyps.

### Non-specific symptoms at primary diagnosis of FAP

The commonest non-specific symptom was abdominal pain, found in 16% (21/129) of patients. It was most common in patients between 40 and 50 years of age, 30% of whom had abdominal pain as predominant symptom (6/20, figure 1). Abdominal pain occurred in 21% (19/90) – 31% (11/36) of patients with the predominant manifestation in the colon, 15% (14/96) – 24% (6/25) of patients with rectal predominance and 43% (3/7) with polyps of the small bowel (tables 1 and 2). Fatigue was reported by 14% (18/130), loss of weight by 12% (15/125), and bloating by 10% (13/130). Pain during defecation was only described in 0.8% (1/130). Most patients who developed these symptoms were between 40 and 50 years old. Fatigue was related to colon carcinomas in 31% (11/36) and in 29% (2/7) to a predominant manifestation in the colon, in 29% (2/7) to polyps of the small bowel and in 27% (7/26) to duodenal polyps. Bloating had the highest association to 10–100 colon polyps, which was 29% (2/7), and to colon carcinomas which was 17% (6/36). Loss of weight showed a correlation to carcinomas of the rectum in 29% (5/17), carcinomas of the colon in 17% (6/36), polyps of the small bowel in 29% (2/7) as well as to duodenal polyps in 23% (6/26) (table 1, 2).

It has to be mentioned that most of our patients had no non-specific symptoms on primary diagnosis of FAP, i.e. no abdominal pain (76%, 108/143), no fatigue (78%, 112/143), no loss of weight (77%, 110/143), no bloating (82%, 117/143) and no pain during defecation (90%, 129/143).

### Bleeding and stool disorders at primary diagnosis of FAP

In 59% (84/143) tests for fecal occult blood were positive. Microscopic bleeding was detected in 72% (55/76) of patients between 20 and 40 years old and 50% (23/46) of patients older than 40 years of age. In 90% (81/90) more than 100 colon polyps, in 82% (14/17) rectal carcinomas, in 61% (22/36) colon carcinomas, in 73% (19/26) duodenal polyps and in 72% (28/39) gastric gland polyps could be detected in association to fecal occult blood. Colonic bleeding as an early symptom was present in 52% (74/143). This was found in 68% (52/76) of patients between 20 and 40 years old and in 35% (16/46) of patients older than 40 years. Colonic bleeding was found in 80% (72/90) of patients with more than 100 colon polyps, 82% (14/17) of patients with rectal carcinoma, 64% (61/96) of patients with 10–100 rectal polyps, 64% (25/39) of patients with gastric gland polyps and 64% (25/39) of those with duodenal polyps. 23% (33/143) of our patients suffered from anemia, 21% (16/76) of patients between 20 and 40 years, and 17% (8/46) of patients older than 40 years of age. Anemia was found in 44% (16/36) of patients with colon carcinoma, 35% (11/17) of patients with rectal carcinoma and 43% (3/7) of those with polyps of the small bowel. Diarrhea was reported in 31% (45/143). 42% (32/76) of patients between 20 and 40 years but only in 24% (11/46) older than 40 years suffered from this symptom. Diarrhea showed an association to rectal carcinomas in 65% (11/17), more than 100 colon polyps in 49% (44/90), duodenal polyps in 46% (12/26) and polyps of the small bowel in 43% (3/7) (tables 1 and 2). 13% (19/143) of the patients reported mucous stools, 6% (9/143) constipation and 3% (5/143) alternating bowel habit. These were the least frequent stool and bleeding disorders in our sample (figure 2).

## Discussion

Most patients with FAP (80%) will be detected by more or less specific symptoms of colorectal disorders which lead to further diagnostic evaluation. Prior to the mid-nineties, when there was no FAP register, families of patients with FAP underwent no routine molecular screening, nor any further specific clinical surveillance. The diagnosis of FAP was verified in 29% of patients by molecular diagnostics, which became available for routine clinical use in the 1990s. The disperson of APC mutations in our study was similar to evaluations concerning other patient populations [15]. Our study also demonstrates the importance of clinical FAP registers, with systematic family screening to identify presymptomatic carriers with APC gene mutations [16,17]. Recent evaluations of physicians' awareness of genetic tests for inherited colorectal cancer showed a need for information concerning the availability and appropriate use of these tests [18]. In principle, genetic testing of index patients and their offspring can be carried out without using a register, but the lack of awareness and possibly the financial costs prevent an effective application of existing knowledge and expertise.

Most patients in our hospital were diagnosed between age 20 and 40 (53%). In 75% (57/76) the diagnosis was made clinically. They mainly presented with fecal occult blood (50%), colonic bleeding (68%) and diarrhea (42%). Rectal polyps were present in 98.7% of patients in this age group. For this reason rectoscopy, which is widely available, should be the first-line investigation in symptomatic patients younger than 40 years of age. Complete colonoscopy should be supplemented if symptoms persist without diagnostic findings during rectoscopy. Some patients presented with extracolonic manifestations such as desmoid tumors. They could be detected in 29% of patients between 20 and 40 years old. In two patients with desmoid tumors, FAP was diagnosed incidentally. In these cases especially, rectoscopy should be performed to exclude polyps. Generally, bleeding and stool disorders were found in cases with severe manifestations in the colon and rectum and colorectal carcinoma. There is an ongoing discussion concerning the benefit of colonoscopy vs. sigmoidoscopy for patients less than 40 years of age with non-acute rectal bleeding [19]. In terms of our findings, FAP in patients younger than 40 years old can certainly be diagnosed with rectoscopy or flexible sigmoidoscopy. As no rectal polyps could be detected in 24% of patients older than 40 years of age, and colon carcinoma was found in 50%, we propose colonoscopy in patients of this age who present with bleeding and stool disorders. Although fecal occult blood, colonic bleeding and diarrhea make a colorectal disease highly probable, they are not specific for FAP and are found in other gastrointestinal disorders such as inflammatory bowel disease, irritable bowel syndrome and malabsorption syndromes or infection, which must always be kept in mind [20-22].

Non-specific symptoms were less frequent and occurred in only 0.8–16% of our patients. They were associated with pronounced colorectal manifestations and many polyps of the upper gastrointestinal tract. Non-specific symptoms were commonest in patients over 40 years old. The incidence of colonic carcinoma was 42–50% in this age group. We conclude that non-specific symptoms can imply a pronounced manifestation of FAP which usually affects patients beyond 40 years of age [12]. Non-specific symptoms are present in other malignancies, endocrine or neuromuscular disorders as well [23-25].

In summary, we found no symptoms specific for FAP in our patient population. Yet it was possible to correlate several symptoms with disease in our age groups. In a few cases, extracolonic manifestations were the first noticeable changes. Due to their young age and mostly moderate symptoms, patients younger than 40 years of age with FAP are not easily identifiable in routine gastrointestinal practice. In all patients who present with the gastrointestinal symptoms we describe, the first diagnostic step should be rectoscopy, followed by complete colonoscopy to exclude FAP. Patients over 40 years old in whom inherited colorectal cancer is suspected, even without rectal manifestations, should generally undergo colonoscopy earlier than recommended for the general population. In patients of all age groups genetic testing should be recommended in order to allow presymptomatic testing in their sibs and offspring. Family members with positive tests and all persons at risk from families where the mutation could not be identified must be examined and monitored by clinical registers.

## Competing interests

The author(s) declare that they have no competing interests

## Authors' contributions

RC had the idea for the manuscript, carried out the statistical analysis, collected the details regarding molecular analysis and wrote the manuscript. WB gave information of several patients concerning molecular diagnostics and symptoms and has given final approval of the version to be published. BR checked the manuscript several times for grammer and spelling failures and was involved in revising it critically for important intellectual content. WH is the director of the Department of Surgery at the University of Erlangen where all patients included in the study were treated. He made the data collection of all patients possible. KG selected the patients and collected the symptoms of each patient. He has made substantial contributions to conception and design of the study. All authors read and approved the final manuscript.

## Pre-publication history

The pre-publication history for this paper can be accessed here:



## Figures and Tables

**Table 1:** „Bleeding and stool disorders“, „non-specific symptoms“ and related colorectal manifestations on the primary diagnosis of FAP (n = 143, values in brackets are relative values)

	**colon**						**rectum**							
		
	**10–100 polyps**		**>100 polyps**		**carcinoma**		**no polyps**		**<10 polyps**		**10–100 polyps**		**carcinoma**	
	**n**	**(%)**	**n**	**(%)**	**n**	**(%)**	**n**	**(%)**	**n**	**(%)**	**n**	**(%)**	**n**	**(%)**
**total patients**	7	(100)	90	(100)	36	(100)	14	(100)	25	(100)	96	(100)	17	(100)
**fecal occult blood**	3	(43)	81	(90)	22	(61)	7	(50)	14	(56)	61	(64)	14	(82)
**colonic bleeding**	2	(29)	72	(80)	17	(47)	4	(29)	12	(48)	56	(58)	13	(76)
**diarrhoea**	1	(14)	44	(49)	16	(44)	1	(7)	6	(24)	37	(39)	11	(65)
**anemia**	3	(43)	30	(33)	16	(44)	4	(29)	4	(16)	25	(26)	6	(35)
**abdomial pain**	2	(29)	19	(21)	11	(31)	1	(7)	6	(24)	14	(15)	3	(18)
**loss of strength**	2	(29)	16	(18)	11	(31)	2	(14)	2	(8)	14	(15)	4	(24)
**loss of weight**	1	(14)	14	(16)	6	(17)	1	(7)	3	(12)	11	(11)	5	(29)
**mucous discharge**	2	(29)	17	(19)	7	(19)	1	(7)	3	(12)	15	(16)	2	(12)
**bloating**	2	(29)	11	(12)	6	(17)	1	(7)	3	(12)	9	(9)	2	(12)
**constipation**	-	-	9	(10)	2	(6)	1	(7)	3	(12)	5	(5)	3	(18)
**paradox diarrhoea**	-	-	5	(6)	1	(3)	-	-	1	(4)	4	(4)	2	(12)

**Table 2:** „Bleeding and stool disorders“, „non-specific symptoms“ and related extracolonic manifestations on primary diagnosis of FAP (n = 143, values in brackets are relative values)

	**desmoid**						**gastric polyps**		**duodenal polyps**		**small bowel polyps**	
							
	**abdominal wall**		**mesenteric**		**retroperitoneal**								
	**n**	**(%)**	**n**	**(%)**	**n**	**(%)**	**n**	**(%)**	**n**	**(%)**	**n**	**(%)**
**total patients**	8	(100)	13	(100)	5	(100)	39	(100)	26	(100)	7	(100)
**fecal occult blood**	4	(50)	9	(69)	4	(80)	28	(72)	19	(73)	4	(57)
**colonic bleeding**	4	(50)	9	(69)	4	(80)	25	(64)	15	(58)	2	(29)
**diarrhoea**	3	(38)	4	(30)	1	(20)	16	(41)	12	(46)	3	(43)
**anemia**	2	(25)	1	(8)	-	-	10	(26)	9	(35)	4	(57)
**abdomial pain**	-	-	-	-	-	-	8	(21)	6	(23)	3	(43)
**loss of strength**	1	(13)	-	-	-	-	7	(18)	7	(27)	2	(29)
**loss of weight**	-	-	-	-	-	-	5	(12)	6	(23)	2	(29)
**mucous discharge**	-	-	5	(39)	1	(20)	7	(18)	5	(19)	1	(14)
**bloating**	-	-	-	-	-	-	4	(10)	3	(12)	1	(14)
**constipation**	-	-	-	-	-	-	1	(3)	1	(4)	-	-
**paradox diarrhoea**	-	-	-	-	-	-	-	-	1	(4)	-	-

**Table 3:** Distribution of age related mutations in the APC gene exons of patients which underwent molecular diagnostics (n = 42)

	**age; years**				
			
	**<20**	**20–40**	**>40**	**n**	**%**
**Exon 1**					
**Exon 2**					
**Exon 3**			1	1	2.3
**Exon 4**		2	1	3	7.1
**Exon 5**	2	3	1	6	14.3
**Exon 6**		2		2	4.8
**Exon 7**					
**Exon 8**		1	1	2	4.8
**Exon 9**					
**Exon 10**			1	1	2.3
**Exon 11**					
**Exon 12**					
**Exon 13**		3		3	7.1
**Exon 14**	2	3	1	6	14.3
**Exon 15**	5	7	6	18	42.9
